# Kinetically Trapped Ligand Binding in DNA Tandem Repeats

**DOI:** 10.1021/acs.biochem.5c00640

**Published:** 2026-02-17

**Authors:** Rabia Tahir, Shankar Pandey, Jacob Haller, Elizabeth Colecchia, Philip Yangyuoru, Hanbin Mao

**Affiliations:** † Department of Chemistry and Biochemistry, 4229 Kent State University, Kent, Ohio 44240, United States; ‡ Boyle Health Sciences Center, Seton Hill University, Greensburg, Pennsylvania 15601, United States; § Department of Chemistry, 5336 Northern Michigan University, Marquette, Michigan 49855, United States

## Abstract

Tandem DNA repeats are ubiquitous in the human genome. They often exist in microsatellite and minisatellite domains, serving as targets for transcription factors and small molecules in gene regulation. Investigation of ligand binding to tandem DNA repeats is rather challenging using traditional methods, such as NMR spectroscopy and X-ray crystallography, whose ensemble-averaging nature prevents deconvolution of individual binding events in a dynamic equilibrium. Harnessing the high sensitivity and temporal resolution of single-molecule techniques such as optical tweezers, we interrogated the binding mechanism between netropsin, a DNA minor groove binder with anticancer properties, and individual recognition sites in the adenine-thymine (A-T) DNA repeats. Surprisingly, we found that the binding between netropsin and the A-T DNA repeats favored kinetically trapped states over thermodynamically stable ones. Although kinetic traps are known in the folding of biomolecules, such kinetically trapped misbinding between ligands and biomolecules, particularly in DNA, has not been directly demonstrated until now. Given the widely occurring tandem repeats of proteins and nucleic acids, our study provides the first direct demonstration in DNA that ligand binding to such repeats can enter kinetically trapped states, which may represent a fundamental aspect of ligand–receptor interactions in cells. Our findings therefore offer insights into new molecular binding mechanisms which may modulate subsequent biological activities.

## Graphical abstract

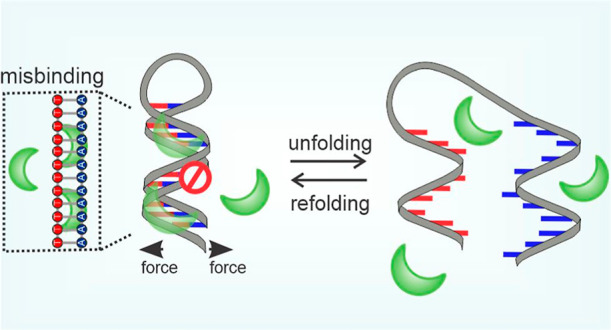

## Introduction

Tandem DNA repeats, particularly in minisatellites and microsatellites, are ubiquitous with 3–8% ^1–3^ occurrence in the human genome.^4^ These regions are important in gene regulation associated with various genetic disorders, including diabetes and Huntington’s disease.^5^ Tandem-repeat instability and repeat expansions have also been implicated broadly in human disease, including several neurological, neurodevelopmental, and cancer-associated pathologies.^3,6^ Gene regulation can start with the binding of transcription factors (TF) to short DNA repeats within gene promoters.^7^ Tandem DNA repeats help to modulate transcription efficacy by two mechanisms. First, multiple DNA repeats allow accommodation of many TF units in the promoter, increasing the modulation efficiency of DNA transcription. Second, repetitive DNA units reduce the entropy penalty associated with TF binding compared to the standalone DNA-TF bindings, increasing the binding rate constant (*k*_on_) while keeping dissociation rate constant (*k*_off_) unaffected.^8^ As a result, the overall binding affinity of TFs to DNA repeats increases, allowing faster and more potent regulation.

Beyond TF proteins, tandem DNA repeats can also serve as binding targets for small-molecule ligands. In the human telomere with a consensus repeat of 5′-TTAGGG, for example, multiple G-quadruplexes can form in the region. Small molecules such as pyridostatin (PDS) with submicromolar affinity can bind to telomeric G-quadruplexes to exert their biological functions.^9,10^ When binders do not have strong affinities with DNA repeats, fast and reversible on–off binding is expected to occur between binders and repetitive DNA fragments, resulting in binding with maximized potency to carry out subsequent biological activities. In the case of small-molecule binders with strong binding affinity to DNA repeats, it is not clear whether the same on–off mechanism exists to optimize the binding efficiency.

Here, we use netropsin, a small-molecule antibiotic first isolated from the bacterium *Streptomyces netropsis*,^11^ to investigate its binding mechanism with DNA repeats. Comprising unfused aromatic rings and basic groups at each terminus (Figure 1A, inset), netropsin assumes a crescent shape to insert into the minor groove of adenine-thymine (A-T) rich duplex DNA.^12,13^ The reported binding affinity of netropsin to double-stranded DNA (dsDNA) typically falls within the micromolar range; however, specific sequences, such as A-T-rich motifs,^14^ exhibit higher affinities (*K*_d_) in the nanomolar range.^15,16^ With its positively charged ends, netropsin is electrostatically attracted to the negatively charged phosphate backbone of DNA strands.^17^ The molecule’s intrinsic twist facilitates its insertion into the DNA minor groove formed by repeating A-T sequences.^16,18,19^ This insertion inhibits enzymes crucial for DNA synthesis,^20,21^ impeding the growth of human breast cancer cells,^22^ as well as Epstein–Barr Virus (EBV) in the lymphoma cells^23^ and inducing programmed cell death.^21,23^ We selected netropsin as a model ligand because not only it provides a simple and well-defined system for probing ligand-DNA interactions in tandem repeats,^17^ but also has served as a foundational parent scaffold for a wide class of minor-groove binding ligands.^24–26^

**Figure 1:**
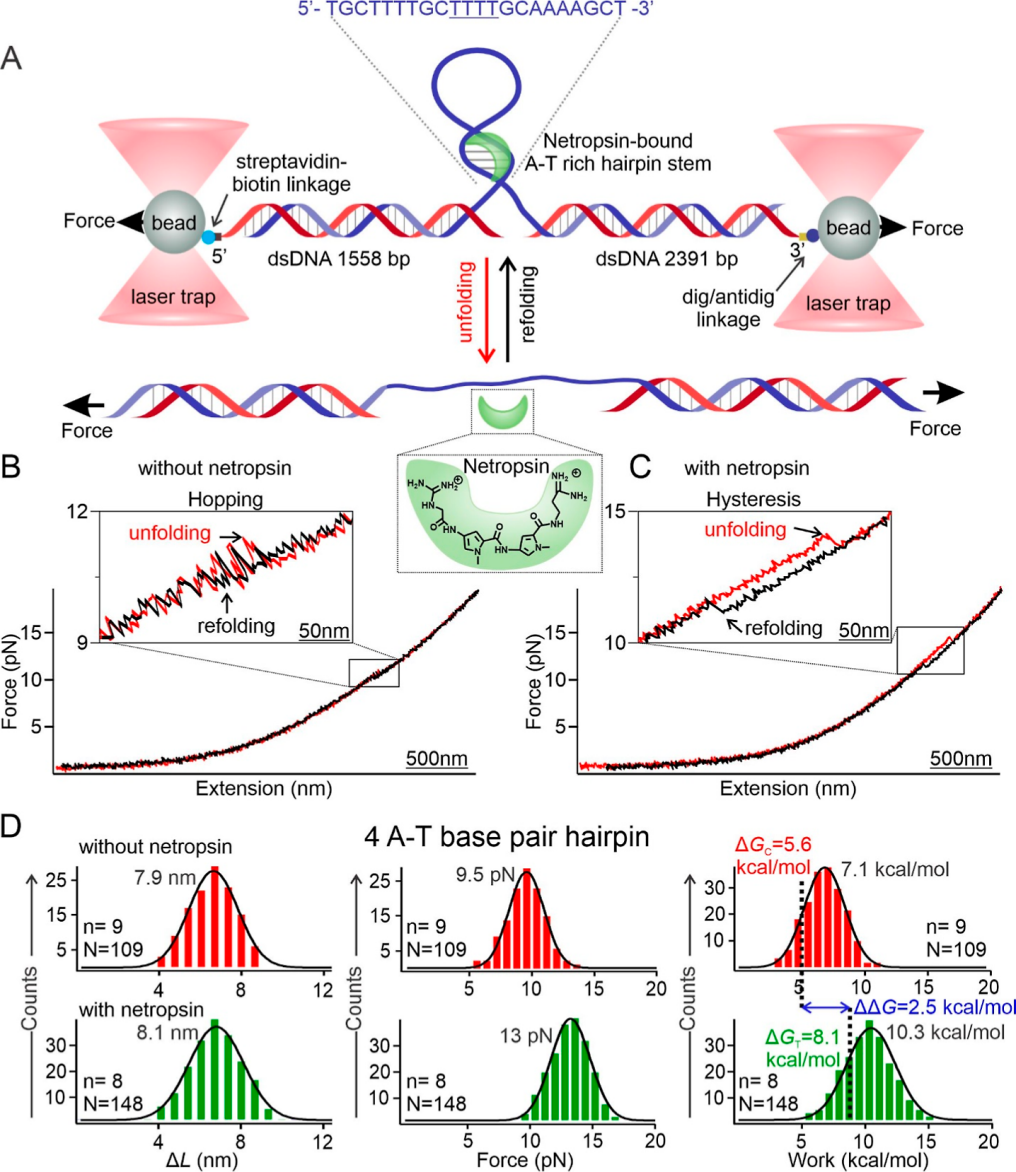
Mechanical unfolding of DNA hairpins with and without netropsin binding. (A) Schematic of the optical-tweezers setup used to mechanically unfold and refold a DNA hairpin containing four A-T base pairs and a tetraloop (underlined in the sequence), with netropsin bound in the minor groove. Dig and antidig represent digoxigenin and antidigoxigenin, respectively. (B) Representative force vs extension (*F*–*X*) curves for unfolding (red) and refolding (black) of the DNA hairpin in the absence of netropsin, measured in 100 mM KCl and 10 mM Tris buffer (pH 7.4) at 23 °C. The inset highlights hopping events between folded and unfolded states (see Materials and Methods for details). (C) *F*–*X* curves for the same hairpin in the presence of 100 nM netropsin under identical buffer conditions. The inset shows a hysteresis region characteristic of ligand-bound *F*–*X* curves (see Materials and Methods for details). (D) Histograms for change-in-contour-length (Δ*L*), unfolding force, and unfolding work for the 4 A-T base pair containing hairpin in the absence (top) and presence (bottom) of netropsin. Vertical dotted lines indicate the Δ*G* values independently calculated using the Jarzynski equality, which represent the change in unfolding free energies. Δ*G*_C_, Δ*G*_T_, and ΔΔ*G* represent the change in free energy associated with hairpin unfolding without netropsin, with netropsin, and the difference between the two, respectively. Solid curves represent Gaussian fits and each histogram peak corresponds to the mean Δ*G* value obtained from the fitted distribution. Numbers indicate mean values. *N* and *n* represent the total number of data points and the number of molecules, respectively.

It is rather challenging for conventional structure-determining approaches such as NMR, X-ray crystallography, and circular dichroism (CD)^27,28^ to distinguish individual units in the tandem A-T DNA repeats during their interactions with the netropsin molecules. Single-molecule techniques such as optical tweezers^29^ offer superior sensitivity to scrutinize the behavior of each DNA repeat unit. In this work, we used optical tweezers to investigate the intricate binding mechanism between A-T DNA repeats and netropsin. Surprisingly, we revealed the presence of kinetically trapped binding (i.e., misbinding) between netropsin molecules and A-T repeats. To the best of our knowledge, such misbound states have not been observed previously in biomolecular binding processes. They resemble kinetically trapped intermediates often observed in biomolecular folding.^30,31^ Our results therefore provide a new binding mechanism for tandemly arranged biomolecular receptors, which are present not only in nucleic acids but also in proteins inside cells.^32^ Our work therefore contributes valuable insights into both fundamental biomolecular interactions and potential therapeutic applications.

## Materials and Methods

### Materials

All the DNA oligonucleotides used in the experiments were purchased from IDT (Integrated DNA Technologies, Coralville, IA, USA), and enzymes were purchased from NEB (New England Biolabs, Ipswich, MA, USA). Both the streptavidin-coated polystyrene beads (1.76 μm) and antidigoxigenin antibody-coated polystyrene beads (2.32 μm) were obtained from Spherotech (Lake Forest, IL, USA). Netropsin dihydrochloride was purchased from Biosynth International (Louisville, KY, USA). All other chemicals and reagents were purchased from Sigma-Aldrich or Fisher Scientific and were used without further purification unless otherwise stated.

### Preparation of DNA Hairpin Constructs for Single-Molecule Mechanical Unfolding Experiments

The DNA oligonucleotide sequences to prepare the A-T-rich DNA hairpin constructs are provided in Table S1. Detailed protocols for preparing the DNA constructs can be found in Supporting Information section S3 and Figure S1. To synthesize the DNA hairpin construct, 5′-phosphorylated DNA oligonucleotides with repeating A-T sequences in the hairpin stem were employed. These oligonucleotides were designed to form a core structure consisting of a stem and loop through annealing. The resulting hairpin structure was then ligated to two double-stranded 5′-biotinylated 1558 base pair and 3′-digoxigenin (dig)-labeled 2391 base pair DNA handles (see dsDNA handle preparation in Supporting Information section S2).

### Single-Molecule Mechanical Unfolding Experiments

The optical tweezers setup used for single-molecule experiments has been previously described.^33^ The single-molecule platform comprises a single-stranded A-T-rich DNA sequence that can fold into a DNA hairpin structure, which is flanked by two double-stranded DNA (dsDNA) handles (Figure 1A, see Supporting Information section S2 for preparation). The DNA hairpin constructs with biotin and digoxigenin labels at the ends were incubated with streptavidin-coated polystyrene beads (1.76 μm diameter) for 15 min to allow the binding of the DNA to the beads via the biotin–streptavidin interaction. The DNA-bound beads were then introduced into the top channel of a four-channel microfluidic chamber, while antidigoxigenin-coated beads (2.32 μm diameter, suspended in 10 mM Tris and 100 mM KCl, pH 7.4) were injected into the bottom channel. These two channels were connected to the middle reaction channel through two capillary tubes (King Precision Glass, Inc., CA, inner diameter 0.025 ± 0.010 mm). The middle channel was partitioned into two subchannels using Parafilm. While the top subchannel contained 10 mM Tris buffer supplemented with 100 mM KCl (pH 7.4), the bottom subchannel contained 100 nM netropsin in the same buffer. Using two laser foci, the two types of beads were separately trapped in the middle reaction channel. The two beads were then brought closer to each other by a laser-steering mirror. This allowed the DNA hairpin construct to be tethered between the beads via digoxigenin/antidigoxigenin and biotin/streptavidin linkages (Figure 1A). By moving two trapped beads away from each other, mechanical force was applied onto the DNA hairpin with a loading rate of approximately 5.5 pN/s (in the 10–30 pN range). After reaching the force required to unfold the DNA hairpin, the force was reduced to zero at the same loading rate, allowing the DNA hairpin to refold before the next force-ramp cycle. A single DNA tether was confirmed by observing a plateau at around 65 pN in the force vs extension (*F*–*X*) curve for the tethered DNA.^34^ The rupture event observed in the force vs extension (*F*–*X*) curve indicated the unfolding of the hairpin structure formed in the A-T-rich sequence. Data were recorded using a LabVIEW program (National Instruments, Austin, TX) and custom MATLAB (The MathWorks, Natick, MA) scripts were employed for data acquisition and processing. The processed data were subsequently analyzed using IGOR Pro v6.37 (WaveMetrics, Portland, OR). Custom IGOR programs were utilized to measure the hairpin unfolding force, change-in-contour-length (Δ*L*, see Supporting Information section S5 for details), and unfolding work (see eq S3 in section S8). These measurements were presented as histograms (Figure 1D for example) and further processed to retrieve relevant parameters. The *F*–*X* curves and the corresponding histograms for all hairpins are presented in Figures S2–S10.

*F*–*X* curves were collected in both the buffer subchannel and the target subchannel containing 100 nM netropsin in 10 mM Tris buffer containing 100 mM KCl at pH 7.4. In the buffer subchannel, a hopping phenomenon in the *F*–*X* curve was observed (Figure 1B), indicating rapid unfolding and refolding of the DNA hairpin^35^ at approximately the same force in the absence of the ligand. In contrast, the *F*–*X* curves in the netropsin channel displayed a hysteresis region (Figure 1C), where unfolding occurred at a higher force than refolding, indicating an increased mechanical stability of the hairpin in the presence of netropsin. The unfolding and refolding *F*–*X* traces did not follow the same trajectory, giving rise to hysteresis,^36^ which is indicative of netropsin binding to the A-T repeats in the hairpin (Figure 1C). The applied force in our assay is a controlled probe, not a physiological mimic: it reveals binding registers by unfolding the hairpin but does not alter the intrinsic binding modes. All ligand-DNA interactions are detected after the molecule has refolded at zero force.

A series of hairpin sequences with different numbers of A-T base pairs in the hairpin stem were synthesized (see Supporting Inforamtion section S3 for details) to investigate the pattern of netropsin binding. The initial hairpin consisted of 4 A-T base pairs in its stem, which progressively increased to 8, 12, 16, and 20 A-T base pairs in the hairpin stem.

## Results and Discussion

### ΔΔ*G* Values Correspond to the Number of Netropsin Molecules Bound to the A-T DNA Repeats

The thermodynamics of netropsin binding were evaluated by the difference in the change in free energy (ΔΔ*G*) between the ligand-bound (Δ*G*_T_) and ligand-free (Δ*G*_C_) states of the DNA hairpin (Figure 2A). Leveraging the Jarzynski equality, eq 1,^37^ a principle that relates the change in free energy between a two-state system in a nonequilibrium process, we determined the unfolding free energies for ligand-bound (Δ*G*_T_) and ligand-free hairpins (Δ*G*_C_).

1 $${\Delta G}_{unfold}=-k_{B}Tln\sum_{i=1}^{N}\frac{1}{N}\exp\left(-\frac{w_{i}}{k_{B}T}\right)$$

where *k*_B_ is the Boltzmann constant, *T* is the absolute temperature, *N* is the number of experimental repetitions, and *W*_*i*_ is the nonequilibrium work done to unfold the DNA hairpins.

**Figure 2:**
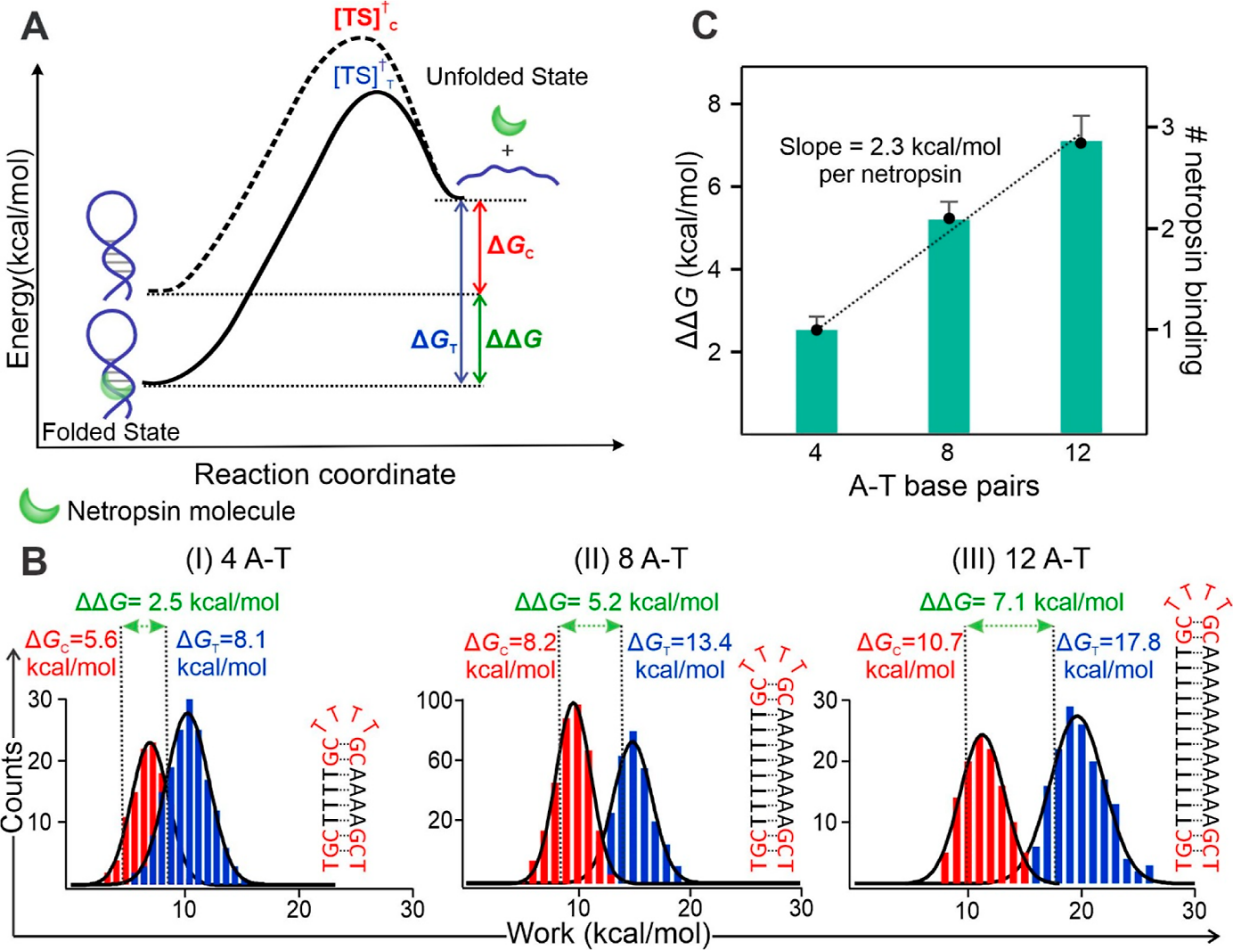
Estimation of netropsin-DNA interactions by difference in free energy change of unfolding (ΔΔ*G*). (A) Schematic energy landscape illustrating the free energy changes associated with unfolding a ligand-free hairpin (dotted curve, Δ*G*_C_) and a ligand-bound hairpin (solid curve, Δ*G*_T_). [TS]^†^_C_ and [TS]^†^_T_ indicate the respective transition states. (B) Unfolding work histograms for hairpins containing four (I), eight (II), and 12 (III) A-T base pairs in the stem (sequences shown to the right of each panel). Red and blue distributions correspond to ligand-free and ligand-bound (100 nM netropsin under the same conditions as described in Figure 1) conditions, respectively. Vertical dotted lines indicate the Δ*G* values independently calculated using the Jarzynski equality, which represent the change in unfolding free energies. Δ*G*_C_ and Δ*G*_T_, with the differences between them representing ΔΔ*G* values corresponding to the binding of one (left panel), two (middle), and three (right) netropsin molecules. Solid curves represent Gaussian fits and each histogram peak corresponds to the mean Δ*G* value obtained from the fitted distribution. (C) Plot of ΔΔ*G* vs the number of A-T base pairs for the hairpins containing four, eight, and 12 A-T base pairs. In comparison, the number of bound netropsin molecules is shown on the right *y*-axis. The data exhibit a strong linear correlation (*R* ^2^ = 0.99), with a slope of 2.3 kcal/mol per netropsin molecule. Error bars represent standard deviations from at least four individual hairpin measurements.

We then calculated the ΔΔ*G* value to quantify the difference in free energy change during the transition from the ligand-bound to the ligand-free state using eq 2 ^38^

2 $$\Delta \Delta G={\Delta G}_{T}-{\Delta G}_{C}$$

The calculated ΔΔ*G* value for the 4 A-T base pair containing hairpin, which is expected to host a single netropsin binding site^17,39–41^ was found to be 2.5 kcal/mol (Figure 2B, left). The dissociation constant (*K*_d_), calculated (see eq S5 in Section S8) from this ΔΔ*G* was 1.47 × 10^–2^ M (see Supporting Information Table S2 for *K*_d_ values for all hairpin constructs), which is higher than previously reported values.^15,18,42^ Under mechanical tension, the DNA hairpin does not remain fully folded but may exist as a mixture of fully folded and partially frayed conformations, where 1–3 base pairs transiently open in the hairpin stem.^35^ This force-induced fraying, analogous to thermal breathing, affects only terminal base pairs while not altering the spacing between internal binding sites. Since netropsin relies on a well-formed minor groove to bind, it exhibits reduced binding affinity under this force-induced fraying, contributing to the higher dissociation constant (*K*_d_) observed here.

Given that a minimum of 4 A-T base pairs is required^17,39–41^ for the binding of one netropsin molecule, the ΔΔ*G* value of 2.5 kcal/mol could be used as the baseline value for the binding of one netropsin molecule. To more accurately determine this ΔΔ*G* value, next, we evaluated the netropsin binding to the 8 (Figure 2B, middle) and 12 A-T base pair containing hairpins (Figure 2B, right) using the same mechanical unfolding approach. After we plotted all ΔΔ*G* values (Figure 2C), we found that a linear relationship existed between the ΔΔ*G* and the length of A-T stems. From the linear fitting, we obtained a slope of 2.3 kcal/mol, which not only matches well with the 2.5 kcal/mol free energy increase from the 4 A-T base pair containing hairpin obtained above, but also indicates that each additional netropsin binding requires four more A-T base pairs in the hairpin stem.

To further confirm that every set of four A-T base pairs binds one netropsin molecule,^17,39–41^ we interspersed guanine-cytosine (G-C) base pairs as spacers at different positions on the 12 A-T base pair containing DNA hairpin construct (Figure 3A–C). Notably, the results obtained from these three constructs yielded ΔΔ*G* values (7.1, 7.3, and 7.2 kcal/mol, see Figure 3A–C, bottom panel) consistent with those observed for the binding of three netropsin molecules (Figure 2B, right, 7.1 kcal/mol). Consistent with this interpretation, control experiments on DNA hairpin containing only isolated two-A-T- pair motifs show no detectable netropsin binding (Figure S13). Together with the G-C spacer results in Figure 3, these data confirm that stable binding of one netropsin molecule requires a minimum of four consecutive A-T base pairs.

**Figure 3:**
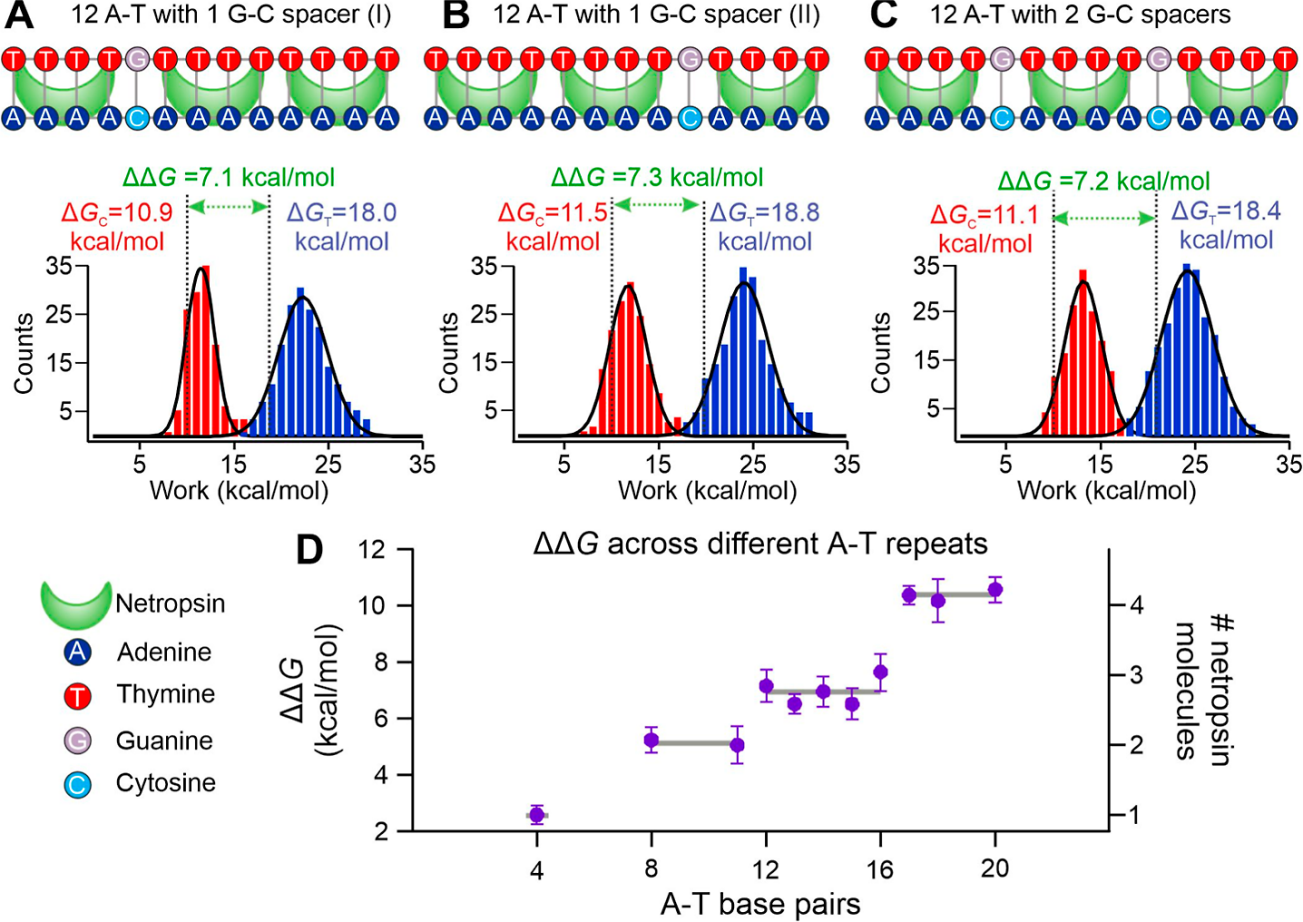
Every four A-T base pairs can accommodate one netropsin molecule. (A–C) Top: schematic representations of DNA hairpins containing 12 A-T base pairs interrupted by one or two G-C base pair spacers to create discrete netropsin binding sites. Bottom: corresponding histograms of unfolding work for each hairpin in the absence (red) and presence (blue) of 100 nM netropsin under the same buffer conditions as in Figure 1. Vertical dotted lines indicate the change in the unfolding free energy in control (Δ*G*_C_, red) and ligand-bound (Δ*G*_T_, blue) conditions independently calculated using the Jarzynski equality, with corresponding ΔΔ*G* values (difference in the change in free energy due to netropsin binding) for all DNA hairpin constructs. Solid curves represent Gaussian fits and each histogram peak corresponds to the mean Δ*G* value obtained from the fitted distribution. (D) Plot of ΔΔ*G* versus the number of A-T base pairs in hairpin stems. The corresponding number of bound netropsin molecules is shown on the right *y*-axis. Horizontal lines indicate average ΔΔ*G* values for different data clusters of A-T base pair lengths. Error bars depict standard deviations from at least four independent hairpin measurements (*n* = 4).

### Steric Hindrance Limits Netropsin Binding to DNA Repeats Beyond 12 A-T Base Pairs

Next, we investigated the binding of netropsin to DNA sequences containing more than 12 A-T base pairs. When the number of A-T repeats was increased one by one from 13 to 15, the ΔΔ*G* values were 6.5, 7.0, and 6.5 kcal/mol for 13, 14, and 15 A-T base pairs, respectively (Figure 3D). All these values are consistent with the expected value (2.3 kcal/mol × 3 = 6.9 kcal/mol) to host three netropsin molecules, which is expected since one netropsin molecule binds to four A-T base pairs. Surprisingly, we observed a ΔΔ*G* of 7.6 kcal/mol for the 16 A-T base pair hairpin (Figure 3D), suggesting it could only host three netropsin molecules rather than the expected four (i.e., (16 A-T)/(4 A-T) = 4 binding sites). Such a result suggests steric hindrance may play a role in limiting the binding of maximally allowed netropsin molecules. To test whether spatial adjustments could alleviate this steric hindrance, one additional A-T base pair was added to the 16 A-T base pair hairpin. Indeed, we found a ΔΔ*G* value of 10.7 kcal/mol, indicating the binding of the fourth netropsin in the 17 A-T base pair containing hairpin (assuming ∼2.3 kcal/mol per netropsin binding event, see Figure 3D). Binding of four netropsin molecules was also confirmed in the 18 (ΔΔ*G* = 10.5 kcal/mol) and 20 (ΔΔ*G* = 10.9 kcal/mol) A-T base pair containing hairpins (Figure 3D). We reasoned that the addition of extra A-T base pairs provides sufficient space to accommodate an additional netropsin molecule, confirming that steric hindrance is indeed the cause for limited netropsin binding in the 16 A-T base pair hairpin. It is notable that the 20 A-T base pair containing hairpin binds to four rather than the expected five netropsin molecules, again indicating steric hindrance as a limiting factor in the ligand binding.

### Kinetically Trapped Misbinding States Lead to the Steric Hindrance Effect

The observed steric hindrance in netropsin binding could be explained by the formation of a kinetically trapped (or “misbound”) netropsin-DNA complex, in which the ligand molecule binds slightly out of register with the four consecutive A-T base pairs (Figure 4A). This misbound molecule blocks nearby vacant A-T sites, preventing additional ligands from recognizing and binding to the maximally allowed binding sites (Figure 4A­(II)). Alternatively, during the experimental time scale, the netropsin binding to the available A-T base pairs may be too slow to allow recognition and binding to the DNA in a timely manner (Figure 4A­(III)).

**Figure 4:**
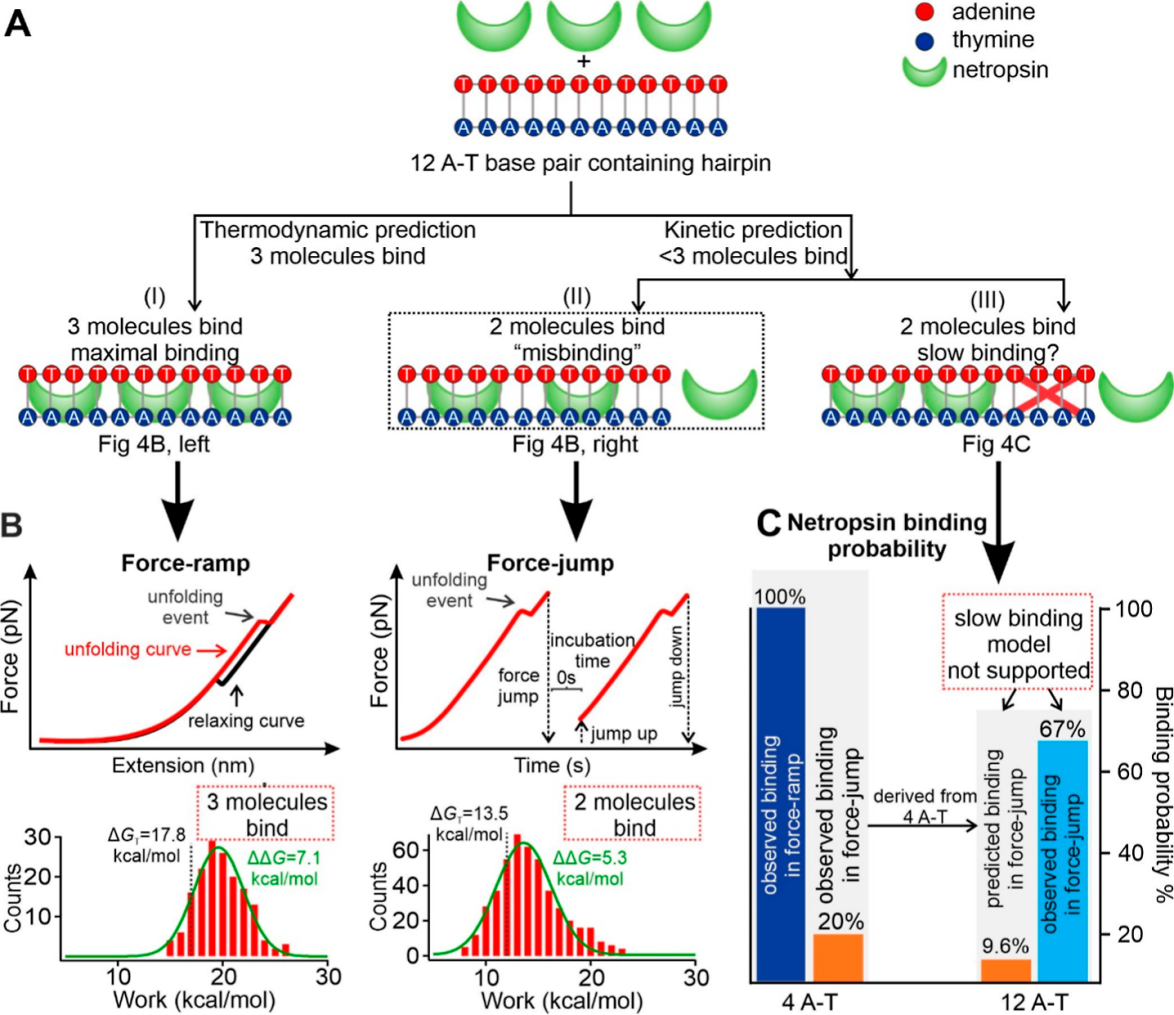
Kinetically trapped binding state of netropsin molecules to tandem A-T DNA base pairs. (A) Models illustrating netropsin binding modes for a hairpin containing 12 A-T base pairs. (B) Top: experimental schematics for force-ramp (left) and force-jump (right) single-molecule assays used to probe netropsin-DNA interactions. Bottom: corresponding unfolding work histograms for the 12 A-T base pair hairpin in the presence of 100 nM netropsin with measured ΔΔ*G* = 7.1 kcal/mol (∼3 bound netropsin molecules) under force-ramp (left) and 5.3 kcal/mol (∼2 bound netropsin molecules) under force-jump (right) conditions. Vertical dotted lines indicate the Δ*G* values independently calculated using the Jarzynski equality, which represent the change in unfolding free energies in the presence of netropsin (Δ*G*_T_). The ΔΔ*G* values were calculated after comparing with Δ*G*_C_ values (unfolding free energy change in the absence of netropsin, 10.7 kcal/mol under force-ramp and 8.2 kcal/mol under force-jump conditions, see the main text). Solid curves represent Gaussian fits and each histogram peak corresponds to the mean Δ*G* value obtained from the fitted distribution. Experiments were performed under 100 mM KCl, 10 mM Tris buffer (pH 7.4) at 23 °C. (C) Bar graphs comparing predicted and observed netropsin binding probabilities for 4 A-T and 12 A-T base pair hairpins under force-ramp or force-jump conditions.

To differentiate these two mechanisms, we measured the kinetics of netropsin binding to the A-T repeats. To this end, we performed force-jump experiments^38^ (Figure 4B, right) using the 12 A-T base pair containing hairpin (see Supporting Information section S6 for details). First, the hairpin was fully unfolded by ramping up to a sufficiently high force (>20 pN), followed by a rapid relaxation to 0 pN to allow refolding. Next, the force was rapidly jumped to a level below the unfolding force of the hairpin (e.g., 5 pN, this jumping step is to reduce the chance of refolding during the slow force-ramp procedure at low force regimes), followed by continuous force-ramp (5.5 pN/s) to reveal whether the hairpin had refolded during the 0 pN incubation. The refolded hairpin during previous incubation at 0 pN would result in an unfolding event in the *F*–*X* curve. Force-jump experiments revealed that the 12 A-T hairpin, which could accommodate up to three netropsin molecules in the slow force-ramp procedure [loading rate: 5.5 pN/s with 30 s incubation at 0 pN, see Figure 4B; ΔΔ*G* = 7.1 kcal/mol, which is calculated from the difference between Δ*G*_C_ (10.7 kcal/mol, see Figure 2B) and Δ*G*_T_ (17.8 kcal/mol, see Figure 2B)], now bound only two netropsin molecules under rapid force-jump conditions (ΔΔ*G* = 5.3 kcal/mol, calculated from the difference between Δ*G*_C_ (8.2 kcal/mol, Figure S11B) and Δ*G*_T_ (13.5 kcal/mol, Figure 4B)). This result indicated that, on average, two out of three sites in the 12 A-T base pair containing hairpin were occupied by the netropsin under force-jump conditions (i.e., ∼67% binding probability).

As a comparison, we also evaluated the probability of netropsin binding to the 4 A-T base pair containing hairpin which has a single netropsin binding site. The force-jump experiments showed that the netropsin binding probability decreased to 20% (Figure 4C, 2^nd^ bar from left). In contrast, in slow force-ramp experiments with 30 s incubation time at 0 pN, a 100% binding probability was observed for the 4 A-T base pair hairpin (Figure 4C, leftmost bar). Based on the 20% netropsin binding probability observed in the 4 A-T base pair containing hairpin in the force-jump experiment, we calculated the expected probability of two netropsin-occupied sites in the 12 A-T base pairs (which can host up to 3 netropsin molecules under slow force-ramp procedure, see Figure 4B, bottom left panel) under the force-jump condition. For each of the three possible cases where two netropsin molecules are bound to the three available sites (i.e., bound–bound-unbound, bound-unbound-bound, or unbound–bound–bound), the probability was determined as 20% (for one of the netropsin-bound sites) × 20% (for another netropsin-bound site) × 80% (for the probability of an unbound site) = 3.2%. Since there are three such cases (i.e., bound–bound-unbound, bound-unbound-bound, or unbound–bound–bound), the total binding probability of two netropsin molecules in the 12 A-T base pair containing hairpin is 3 × (20 % × 20 % × 80%) = 9.6% (Figure 4C, the third bar from left). Compared to the experimental observation in which binding of two netropsin molecules had 67% probability (Figure 4C, rightmost bar), the predicted probability is much lower (9.6%). Since this prediction is based on the fully registered binding (i.e., the maximum binding possibility, Figure 4A, top) between one netropsin and every 4 A-T base pairs, the out-of-register binding (Figure 4A, middle) must play a significant role. These out-of-register binding events prevented the maximal binding between one netropsin and every four A-T base pairs, which represents a kinetically trapped misbinding mechanism (Figure 4A, middle panel). Consistent with this, force-jump experiments on the 12 A-T hairpin containing two G-C spacers, which separate the 12 A-T sequence into three independent binding sites, yielded a ΔΔ*G* of 7.1 kcal/mol. This value corresponded to the binding of three netropsin molecules (Figure S12), confirming that the reduced occupancy observed in continuous A-T repeats (Figure 4) arises from kinetic trapping rather than from an intrinsic limitation in binding capacity.

## Conclusion

In summary, using single-molecule mechanical unfolding in an optical tweezers instrument, we quantified the change in free energy associated with the unfolding of A-T rich DNA hairpins in the presence and absence of netropsin molecules (ΔΔ*G*). Such a measurement revealed that each netropsin can bind to four tandemly arranged A-T base pairs within the DNA hairpin stem. However, by performing force-jump experiments under rapid kinetic conditions, we observed a systematic decrease in netropsin occupancy for DNA fragments longer than 12 A-T base pairs. This indicates that in continuous A-T repeats, netropsin molecules do not always bind to the canonical 4 A-T base pairs in register. It frequently occupies out-of-register positions that sterically block adjacent available sites. Such misbound configuration generates kinetically trapped states that prevent the sequence from achieving its maximal thermodynamic ligand-binding capacity. Because netropsin dissociates slowly, these suboptimal binding configurations persist as kinetically trapped states, such that ligand occupancy in tandem A-T repeats is determined not only by equilibrium affinity but also by dissociation kinetics (*k*_off_), a factor not accessible in ensemble measurements.

These findings provide the first direct, site-resolved evidence that a minor-groove binder can exhibit nonequilibrium binding behavior within tandem DNA repeats. More broadly our study highlights how kinetic factors and register discrepancy shape ligand-DNA interactions and reveals a binding mechanism relevant to tandemly arranged biomolecular receptors, which occur widely in genome and have broad implications for gene regulation and therapeutic targeting. Because our experiments establish the mechanistic basis of misbinding, a generic phenomenon that is governed by slow *k*_off_ kinetics, in a well-studied model system, they provide the foundation for future studies examining how other DNA-binding ligands or different DNA repeat types exhibit similar or distinct kinetic effects.

## Supplementary Material



## Data Availability

All data will be available upon request.

## Notes

DNA sequences and construct designs for A-T rich hairpin systems; preparation of DNA handles and single-molecule constructs; optical tweezers force-ramping and force-jump experimental protocols; detailed data analysis procedures; representative force–extension curves and unfolding histograms for hairpins of varying A-T lengths and G-C spacers; free-energy and dissociation constant calculations for netropsin binding; control experiments on G-quadruplex hairpins; supplementary figures, tables, and references (PDF)
