# Supplementary material for: Kinetically Trapped Ligand Binding in DNA Tandem Repeats
Source: Biochemistry. 2026 Feb 17;65(7):1025–32. doi: 10.1021/acs.biochem.5c00640 (PMC13063410; doi:10.1021/acs.biochem.5c00640)
Supplement: Supplementary file 1 [file bi5c00640_si_001.pdf]

Kinetically trapped ligand binding in DNA Tandem Repeats

Rabia Tahir, [1] *Shankar Pandey,* [1] *Jacob Haller,* [1] *Elizabeth Colecchia,* [1,2]

Philip Yangyuoru,[3] *Hanbin Mao* [1 *]

[1] Department of Chemistry and Biochemistry, Kent State University, Kent, OH 44240, USA

[2] Boyle Health Sciences Center, Seton Hill University, Greensburg, PA 15601, USA

[3] Department of Chemistry, Northern Michigan University, Marquette, MI 49855

[*] Corresponding author: Hanbin Mao (Tel: +1 330 672 9380; Fax: +1 330 672 3816; Email: hmao@kent.edu)

Table of Contents

S1. Table S1. List of sequences used for preparing A-T rich hairpin constructs……..………S3-S4

S2. Preparation of dsDNA handles for synthesis of single-molecule hairpin constructs…………S4

S3. Synthesis of A-T rich hairpin constructs for single-molecule investigations...................S4-S5

Figure S1. General strategy for the preparation of A-T DNA hairpin constructs….S5

S4. Single-molecule force ramping experiments…………………………………………S6

S5. Data analysis………………………………………………………………………….S6-7

S6. Force-jump experiments………………………………………………………………..S7

S7. Typical force vs. extension curves and histograms for A-T rich hairpins.

Figure S2. 11 A-T base pair hairpin F-X curves and histograms...............................S7

Figure S3. 13 A-T base pair hairpin F-X curves and histograms...............................S8

Figure S4. 14 A-T base pair hairpin F-X curves and histograms...............................S9

Figure S5. 15 A-T base pair hairpin F-X curves and histograms...............................S9-10

Figure S6. 16 A-T base pair hairpin F-X curves and histograms...............................S10

Figure S7. 17 A-T base pair hairpin F-X curves and histograms...............................S11

Figure S8. 18 A-T base pair hairpin F-X curves and histograms.............................S12

Figure S9. 20 A-T base pair hairpin F-X curves and histograms..............................S12-13

Figure S10. Hairpins with G-C spacers typical F-X curves……………………..S13

Figure S11. 12 A-T base pair histograms obtained by force-jump experiment…..S14

Figure S12. Free-energy analysis of netropsin binding to a 12 A-T hairpin with G-C spacers …..S15

Figure S13. Single-molecule unfolding of a poly(A) G-quadruplex (GQ) hairpin in the presence and absence of netropsin…………………………………………………….…..S16

S8. Free energy difference and dissociation constant calculation. ………………………….S17

S9. Table S2. ΔΔG for all hairpins and their respective dissociation constants (K_d) …………S18

S10. References…………………..S19

S1. Table S1. List of sequences used for preparing A-T rich hairpin constructs. Underlined text indicates loop sequence.

Name	Sequence (5’-3’)
BsaI-HF	GTCCCTGCGCGACCCGATGCAGAACGACCG
L-primer 9	CGATGTGATCTTCCTGTCATAAAC
4 A-T base pair hairpin	CGGT CAA ATA CTG TCC TTC TAG TGT AGC TGC **TTTT** GC TTTT GC **AAAA** GCT GCC AGC AAG ACG TAG CCC AGC GCG TC
8 A-T base pair hairpin	CGGT CAA ATA CTG TCC TTC TAG TGT AGC TGC **TT TTT TTT** GC TTTT GC **AA AAA AAA** GCT GCC AGC AAG ACG TAG CCC AGC GCG TC
12 A-T base pair hairpin	CGGT CAA ATA CTG TCC TTC TAG TGT AGC TGC **TTT TTT TTT TTT** GC TTTT GC **AAA AAA AAA AAA** GCT GCC AGC AAG ACG TAG CCC AGC GCG TC
13 A-T base pair hairpin	CGGT CAA ATA CTG TCC TTC TAG TGT AGC TGC **TTT TTT TTTT TTT** GC TTTT GC **AAA AAAA AAA AAA** GCT GCC AGC AAG ACG TAG CCC AGC GCG TC
14 A-T base pair hairpin	CGGT CAA ATA CTG TCC TTC TAG TGT AGC TGC **TTT TTT TTTT TTTT** GC TTTT GC **AAA AAAA AAAA AAA** GCT GCC AGC AAG ACG TAG CCC AGC GCG TC
15 A-T base pair hairpin	CGGT CAA ATA CTG TCC TTC TAG TGT AGC TGC **TTTT TTT TTTT TTTT** GC TTTT GC **AAA AAAA AAAA AAAA** GCT GCC AGC AAG ACG TAG CCC AGC GCG TC
16 A-T base pair hairpin	CGGT CAA ATA CTG TCC TTC TAG TGT AGC TGC **TTTT TTTT TTTT TTTT** GC TTTT GC **AAAA AAAA AAAA AAAA** GCT GCC AGC AAG ACG TAG CCC AGC GCG TC
17 A-T base pair hairpin	CGGT CAA ATA CTG TCC TTC TAG TGT AGC TGC **TTTT TTTT TTTT TTTT T** GC TTTT GC **A AAAA AAAA AAAA AAAA** GCT GCC AGC AAG ACG TAG CCC AGC GCG TC
18 A-T base pair hairpin	CGGT CAA ATA CTG TCC TTC TAG TGT AGC TGC **TTTT TTTT TTTT TTTT TT** GC TTTT GC **AA AAAA AAAA AAAA AAAA** GCT GCC AGC AAG ACG TAG CCC AGC GCG TC

20 A-T base pair hairpin	CGGT CAA ATA CTG TCC TTC TAG TGT AGC TGC **TTTT TTTT TTTT TTTT TTTT** GC TTTT GC **AAAA AAAA AAAA AAAA AAAA** GCT GCC AGC AAG ACG TAG CCC AGC GCG TC
12 A-T base pair hairpin with 1 G-C base pair (marked with red) (I)	CGGT CAA ATA CTG TCC TTC TAG TGT AGC TGC **TTTT G TTTT TTTT** GC TTTT GC **AAAA AAAA C AAAA** GCT GCC AGC AAG ACG TAG CCC AGC GCG TC
12 A-T base pair hairpin with 1 G-C base pair (marked with red) (II)	CGGT CAA ATA CTG TCC TTC TAG TGT AGC TGC **TTTT TTTT C TTTT** GC TTTT GC **AAAA G AAAA AAAA** GCT GCC AGC AAG ACG TAG CCC AGC GCG TC
12 A-T base pair hairpin with 2 G-C base pair (marked with red)	CGGT CAA ATA CTG TCC TTC TAG TGT AGC TGC **TTTT G TTTT C TTTT** GC TTTT GC **AAAA G AAAA C AAAA** GCT GCC AGC AAG ACG TAG CCC AGC GCG TC
Poly(A) G-quadruplex (GQ) hairpin	5’ CGG TAC GGT GTG AAA TAC CGC ACA GAT GCG ACG GTC GAT CTG TGA GGT AGT AG (A)21 TTAGGGTTAGGGATTGGGTTAGGG TTA (A)21 CTA CTA CCT CAC AGA TCG ACC GT GCC AGC AAG ACG TA G CCC AGC GCG TC 3’

S2. Preparation of dsDNA handles for synthesis of single-molecule hairpin constructs.

The two dsDNA handles used to tether various A-T rich hairpins were prepared following the protocol outlined in our previous study (1). Briefly, the 1558 bp DNA handle was synthesized by PCR using pBR322 plasmid (NEB) as the template and two primers: 5’-biotin-TEG-GCA TTA GGA AGC CCA GTA GG and 5’-AAA CCA TAG AGG CTA CAC TAG AAG GAC AGT ATT TG. The PCR product was purified and digested with BsaI-HFv2 enzyme (NEB) to generate a 5’ sticky end where the complementary sticky end of the A-T rich hairpin toehold could ligate.

To prepare the 2391 bp DNA handles, PCR was performed using λ-DNA (NEB) as the template and the two primers: 5’-AAA AAA AAG AGC TCC TGA CGC TGG CAT TCG CAT CAA AG and 5’-AAA AAA AAG GTC TCG CCT GGT TGC GAG GCT TTG TGC TTC TC. The PCR product was then labeled at the 3’ end with digoxigenin-dUTP (Enzo Life Sciences) using terminal transferase (Thermo Fisher) for 8 h at 37°C. The resulting product was then digested with BsaI-HFv2 (NEB) to generate a 5’ overhang, yielding the 2391 bp handle.

S3. Synthesis of A-T rich hairpin constructs for single-molecule investigations.

The detailed protocol is shown in Figure S1. For the construct preparation, the specific hairpin-forming A-T rich sequence with netropsin binding site in the stem was first phosphorylated and then annealed with L-primer 9 (see Table S1 for sequence) at the 5' end and a BsaI-HF sequence (see Table S1 for sequence) at the 3' end via a 95°C to 20°C temperature ramp at 1 °C min^{-1}. The annealed product formed a hairpin structure, which was subsequently ligated to the two non-palindromic double-stranded DNA handles of 1558 bp and 2391 bp, prepared as described above, by using T4 DNA ligase (NEB) at 16 °C for 16 h. The prepared construct was then used to perform optical tweezers experiments.

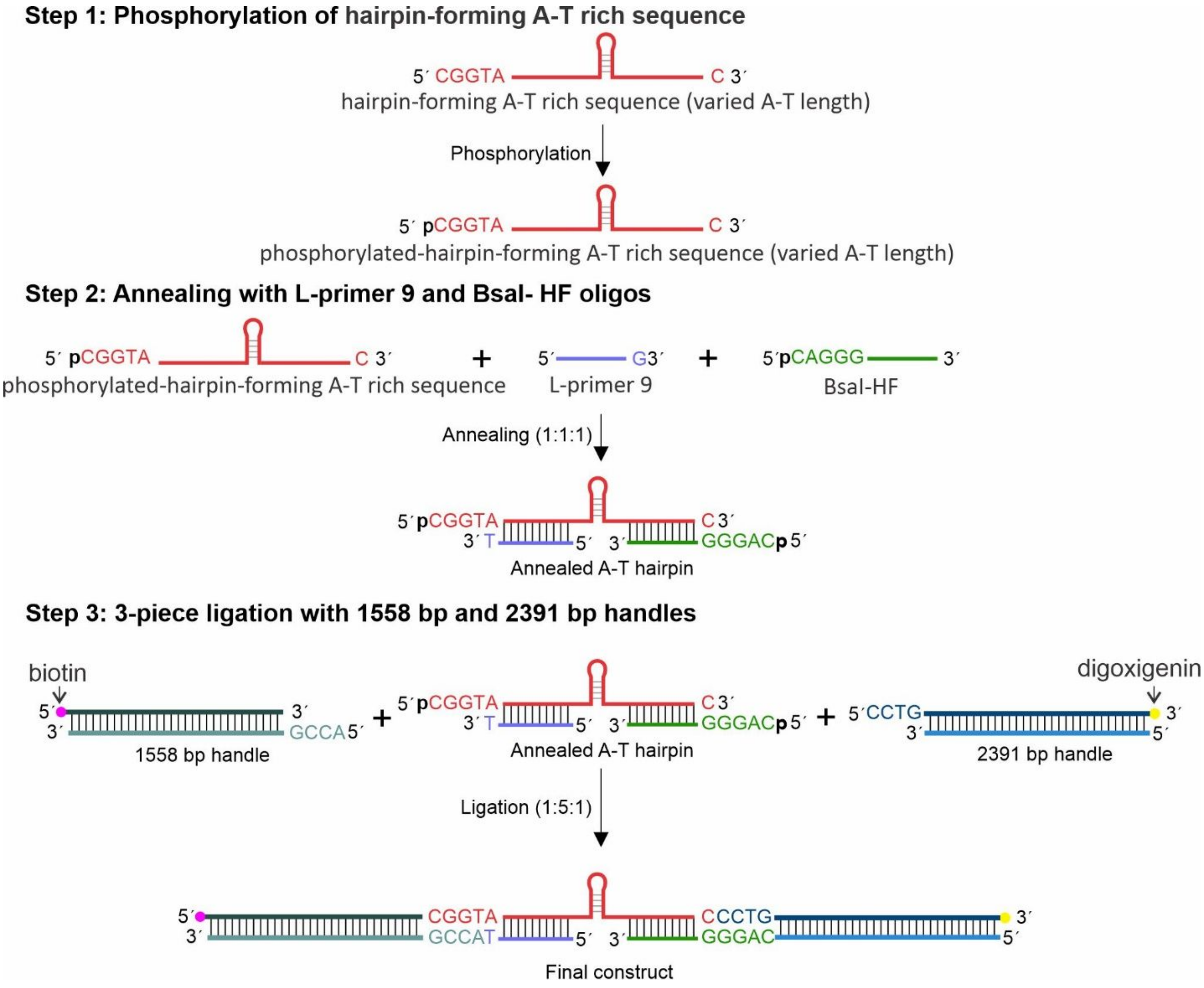

Figure S1. General strategy for the preparation of A-T rich hairpin constructs for mechanical unfolding experiments. Sequences for all the A-T rich hairpin stem and other oligos used are provided in Table S1.

S4. Single-molecule force-ramping experiments

Single-molecule experiments were conducted at 23 °C in 10 mM Tris buffer containing 100 mM KCl (pH 7.4) using a home-built dual-beam optical tweezers setup as previously described (2). Briefly, the prepared A-T rich DNA hairpin constructs labeled with digoxigenin at one end were incubated with anti-digoxigenin-coated polystyrene beads (2.32 µm diameter, Spherotech, IL, USA), which were then immobilized in optical traps. Streptavidin-coated beads (1.76 µm diameter, Spherotech, IL, USA) were trapped separately, and the DNA construct was tethered between the two beads via biotin-streptavidin and digoxigenin-anti-digoxigenin linkages.

One of the two 1064 nm laser foci was fixed while the other was controlled by a steerable mirror. By moving the trapped beads apart using the steerable laser beam, mechanical force was applied to the DNA construct. The force-extension (F-X) curves were recorded using LabVIEW software (National Instruments, Austin, TX) at 1 kHz with a loading rate of 5.5 pN/s (within the 10-30 pN force range). A force plateau around 70 pN indicated the rupture of duplex DNA handles and confirmed the presence of a single DNA tether (4). Unfolding of the A-T rich hairpin was evident as a rupture event in the F-X curve, reflecting disruption of the stem-loop structure.

F-X data were processed using a Savitzky-Golay filter with a 10 ms time constant in MATLAB (The MathWorks, Natick, MA). DNA hairpin unfolding force, change-in-contour-length (ΔL), and unfolding work were extracted from these curves using custom IGOR Pro scripts (WaveMetrics, Portland, OR). The ΔL values were used to verify structural transitions and ligand-binding effects.

S5. Data Analysis

The change in extension (Δx) at a given force (F) was measured by calculating the difference between the stretching and relaxing traces at that force. The resulting Δx was then converted to the change-in-contour-length (ΔL) by applying the worm-like chain (WLC) model (Equation S1) (3,4)

$\Delta x/\Delta L = 1 - 1/2\ (k_B T/FP)^{1/2} + (F/S)$ …….(S1)

where k_B is the Boltzmann constant, T is absolute temperature, P is the persistent length of dsDNA (50.8 nm), and S is the stretching modulus (1243 pN). (4)(5)

S6. Force-jump experiments

To investigate the netropsin-DNA binding kinetics, force-jump experiments were performed as previously described (6). DNA hairpins were first unfolded at a loading rate of 15 pN/s until the rupture event was detected. Immediately after unfolding, the force was rapidly decreased to ~0 pN to allow for possible refolding and ligand binding. After a defined incubation at 0.5 pN, the force was rapidly increased to 5 pN (which is below the rupture force of the hairpin under study), followed by force ramping at a loading rate of 15 pN/s to probe for the hairpin refolding, indicated by a rupture event in the force-extension trace. This setup allowed detection of binding events occurring within ~20 ms. By analyzing the rupture features in the F-X curves (i.e., *ΔΔ*G; see main text), we estimated the number of netropsin molecules binding to the 12 A-T hairpin.

S7: Typical force vs. extension curves and histograms for A-T rich hairpins.

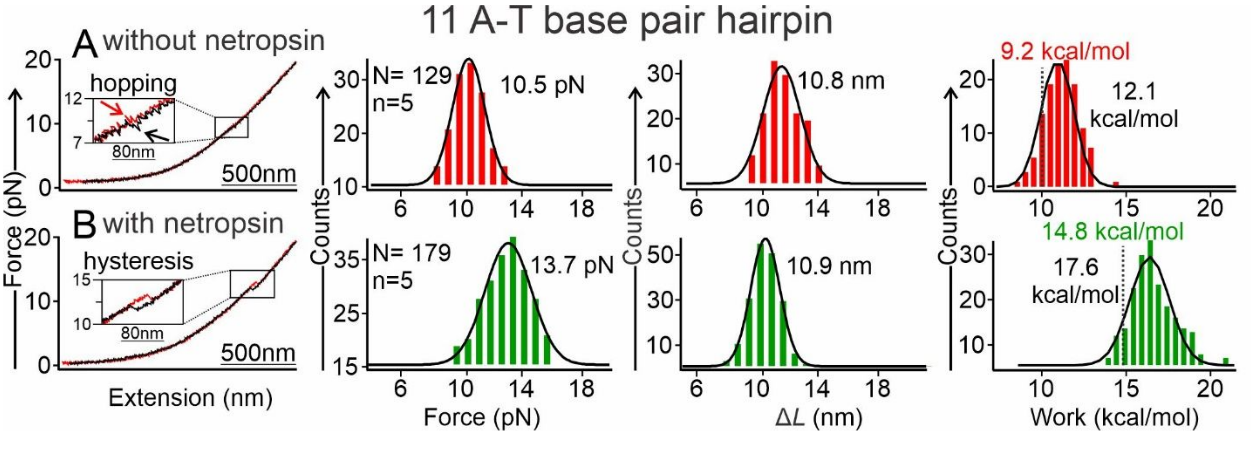

Figure S2. Typical force-extension traces (left) for 11 A-T base pair hairpin in the control channel (A) (100mM KCl, 10 mM Tris buffer, pH 7.4) show hopping (indicated with a dotted box, arrows indicate unfolding (red) and refolding (black) events) and the target channel (B) (same buffer with 100 nM netropsin) showing hysteresis (indicated with a dotted box) with the corresponding histograms of the

unfolding force, change-in-contour-length (ΔL), and work (right). See Table S1 for sequence. Vertical dotted lines indicate the Δ*G* values independently calculated using the Jarzynski equality, which represent the change in unfolding free energies. ΔG_C and ΔG_T represent the change in free energy associated with hairpin unfolding without netropsin and with netropsin, respectively. Solid curves represent Gaussian fits and each histogram peak corresponds to the mean Δ*G* value obtained from the fitted distribution. Numbers indicate mean values. N and n depict the total number of data points and the number of molecules, respectively.

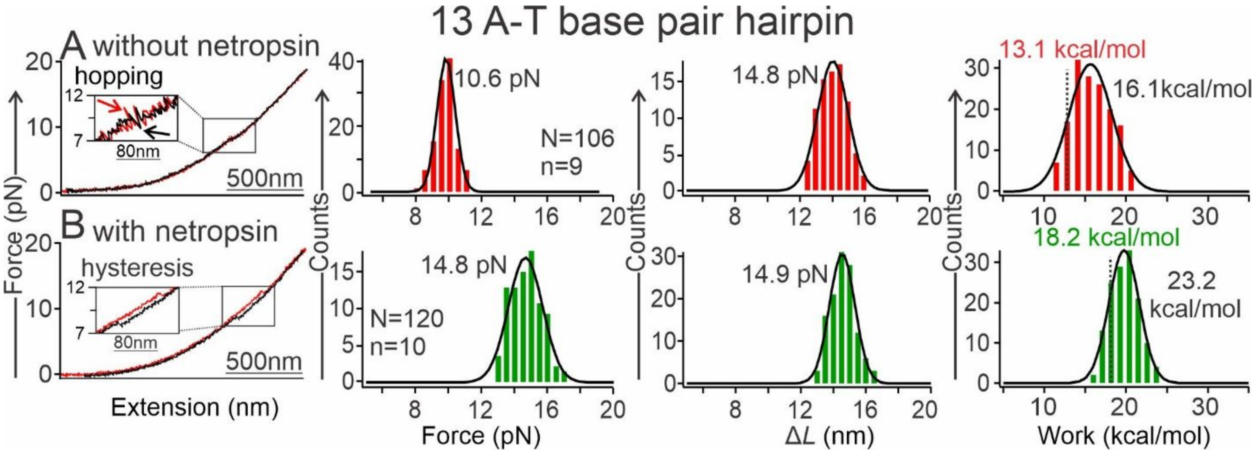

Figure S3. Typical force-extension traces (left) for 13 A-T base pair hairpin in the control channel (A) (100mM KCl, 10 mM Tris buffer, pH 7.4) show hopping (indicated with a dotted box, arrows indicate unfolding (red) and refolding (black) events) and the target channel (B) (same buffer with 100nM netropsin) showing hysteresis (dotted box) with the corresponding histograms of unfolding force, change-in-contour-length (ΔL), and work (right). See Table S1 for sequence. Vertical dotted lines indicate the Δ*G* values independently calculated using the Jarzynski equality, which represent the change in unfolding free energies. ΔG_C and ΔG_T represent the change in free energy associated with hairpin unfolding without netropsin and with netropsin, respectively. Solid curves represent Gaussian fits and each histogram peak corresponds to the mean Δ*G* value obtained from the fitted distribution. Numbers indicate mean values. N and n depict the total number of data points and the number of molecules, respectively.

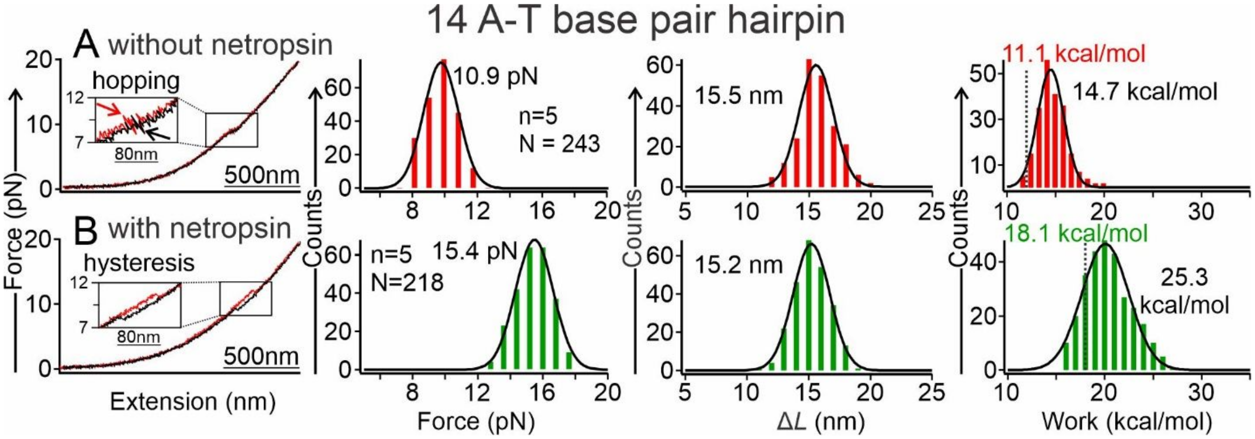

Figure S4. Typical force-extension traces (left) for 14 A-T base pair hairpin in the control channel (A) (100mM KCl, 10 mM Tris buffer, pH 7.4) show hopping (indicated arrows indicate unfolding (red) and refolding (black) events) and the target channel (B) (same buffer with 100nM netropsin) showing hysteresis (indicated with a dotted box) with the corresponding histograms of unfolding force, change-in-contour-length (ΔL), and work (right). See Table S1 for sequence. Vertical dotted lines indicate the ΔG values independently calculated using the Jarzynski equality, which represent the change in unfolding free energies. ΔG_C and ΔG_T represent the change in free energy associated with hairpin unfolding without netropsin and with netropsin, respectively. Solid curves represent Gaussian fits and each histogram peak corresponds to the mean ΔG value obtained from the fitted distribution. Numbers indicate mean values. N and n depict the total number of data points and the number of molecules, respectively.

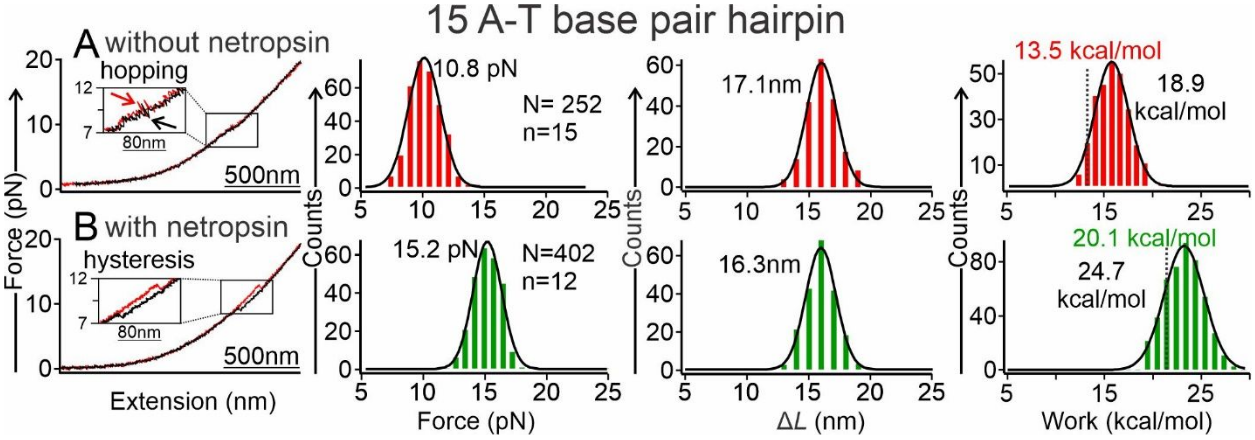

Figure S5. Typical force-extension traces (left) for 15 A-T base pair hairpin in the control channel (A) (100mM KCl, 10 mM Tris buffer, pH 7.4) show hopping (indicated with a dotted box, arrows indicate unfolding (red) and refolding (black) and the target channel (B) (same buffer with 100nM netropsin) showing hysteresis (indicated with a dotted box) with the corresponding histograms of unfolding force, change-in-contour-length (ΔL), and work (right). See Table S1 for sequence. Vertical dotted lines indicate the Δ*G* values independently calculated using the Jarzynski equality, which represent the change in unfolding free energies. ΔG_C and ΔG_T represent the change in free energy associated with hairpin unfolding without netropsin and with netropsin, respectively. Solid curves represent Gaussian fits and each histogram peak corresponds to the mean Δ*G* value obtained from the fitted distribution. Numbers indicate mean values. N and n depict the total number of data points and the number of molecules, respectively.

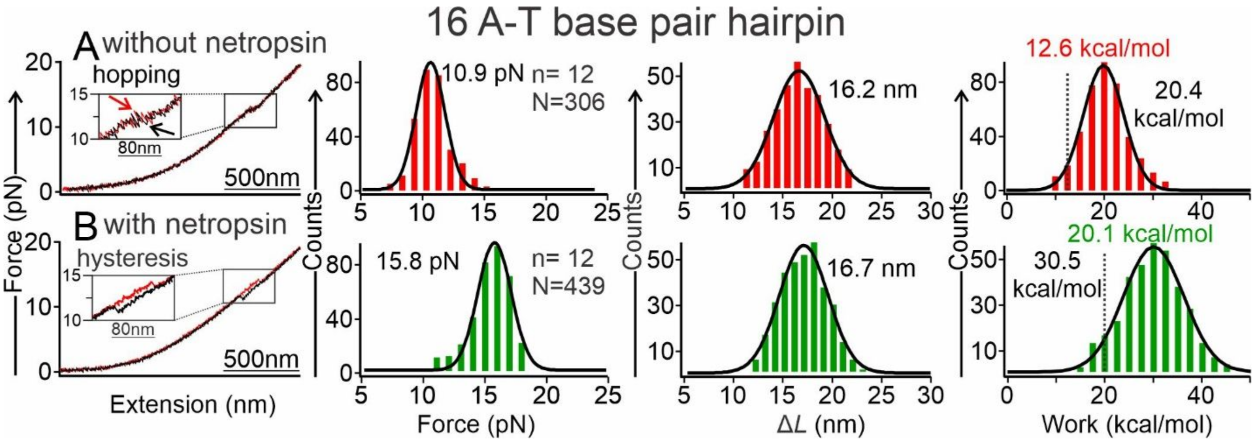

Figure S6. Typical force-extension traces (left) for 16 A-T base pair hairpin in the control channel (A) (100mM KCl, 10 mM Tris buffer, pH 7.4) show hopping (indicated with a dotted box, arrows indicate unfolding (red) and refolding (black) events) and the target channel (B) (same buffer with 100nM netropsin) showing hysteresis (indicated with a dotted box) with the corresponding histograms of unfolding force, change-in-contour-length (ΔL), and work (right). See Table S1 for sequence. Vertical dotted lines indicate the Δ*G* values independently calculated using the Jarzynski equality, which represent

the change in unfolding free energies. ΔG_C and ΔG_T represent the change in free energy associated with hairpin unfolding without netropsin and with netropsin, respectively. Solid curves represent Gaussian fits and each histogram peak corresponds to the mean ΔG value obtained from the fitted distribution. Numbers indicate mean values. N and n depict the total number of data points and the number of molecules, respectively.

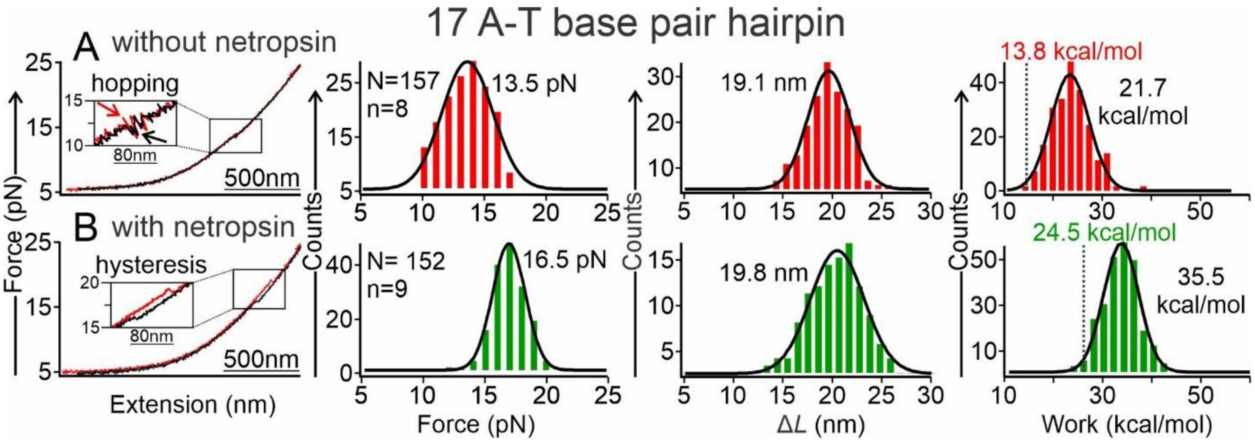

Figure S7. Typical force-extension traces (left) for 17 A-T base pair hairpin in the control channel (A) (100mM KCl, 10 mM Tris buffer, pH 7.4) show hopping (indicated with a dotted box, arrows indicate unfolding (red) and refolding (black) events) and the target channel (B) (same buffer with 100nM netropsin) showing hysteresis (indicated with a dotted box) (B) with the corresponding histograms of unfolding force, change-in-contour-length (ΔL), and work (right). See Table S1 for sequence. Vertical dotted lines indicate the ΔG values independently calculated using the Jarzynski equality, which represent the change in unfolding free energies. ΔG_C and ΔG_T represent the change in free energy associated with hairpin unfolding without netropsin and with netropsin, respectively. Solid curves represent Gaussian fits and each histogram peak corresponds to the mean ΔG value obtained from the fitted distribution. Numbers indicate mean values. N and n depict the total number of data points and the number of molecules, respectively.

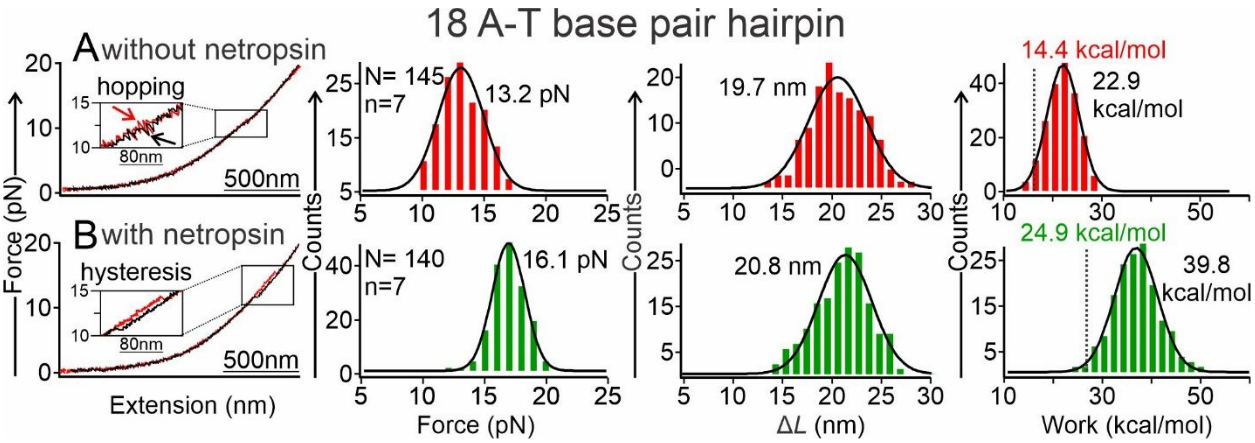

Figure S8. Typical force-extension traces (left) for 18 A-T base pair hairpin in the control channel (A) (100mM KCl, 10 mM Tris buffer, pH 7.4) show hopping (indicated with a dotted box, arrows indicate unfolding (red) and refolding (black) events) and the target channel (B) (same buffer with 100nM netropsin) showing hysteresis (indicated with a dotted box) (B) with the corresponding histograms of unfolding force, change-in-contour-length (ΔL), and work (right). See Table S1 for sequence. Vertical dotted lines indicate the Δ*G* values independently calculated using the Jarzynski equality, which represent the change in unfolding free energies. ΔG_C and ΔG_T represent the change in free energy associated with hairpin unfolding without netropsin and with netropsin, respectively. Solid curves represent Gaussian fits and each histogram peak corresponds to the mean Δ*G* value obtained from the fitted distribution. Numbers indicate mean values. N and n depict the total number of data points and the number of molecules, respectively.

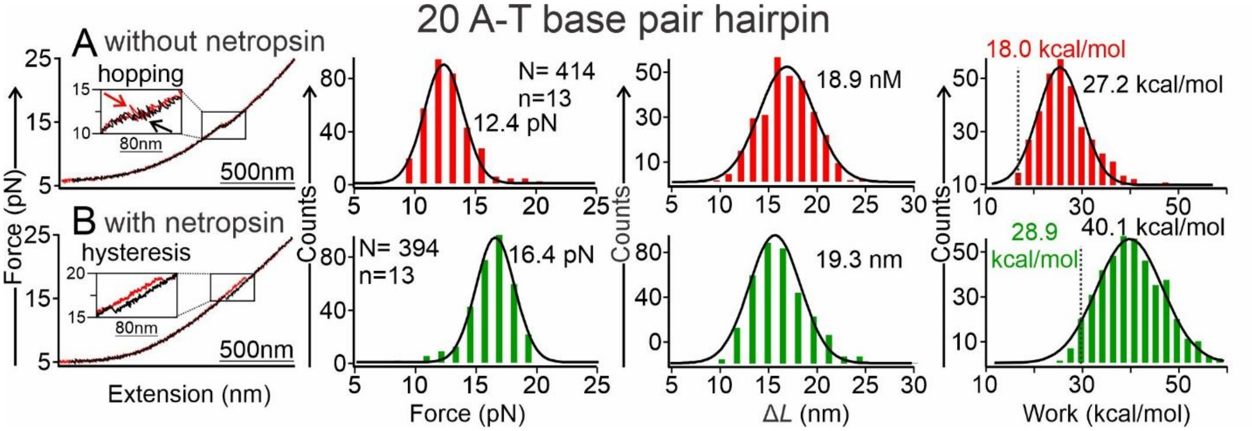

Figure S9. Typical force-extension traces (left) for 20 A-T base pair hairpin in the control channel (A) (100mM KCl, 10 mM Tris buffer, pH 7.4) show hopping (indicated with a dotted box, arrows indicate unfolding (red) and refolding (black) events) and the target channel (B) (same buffer with 100nM netropsin) showing hysteresis (indicated with a dotted box) (B) with the corresponding histograms of unfolding force, change-in-contour-length (ΔL), and work (right). See Table S1 for sequence. Vertical dotted lines indicate the Δ*G* values independently calculated using the Jarzynski equality, which represent the change in unfolding free energies. ΔG_C and ΔG_T represent the change in free energy associated with hairpin unfolding without netropsin and with netropsin, respectively. Solid curves represent Gaussian fits and each histogram peak corresponds to the mean Δ*G* value obtained from the fitted distribution. Numbers indicate mean values. N and n depict the total number of data points and the number of molecules, respectively.

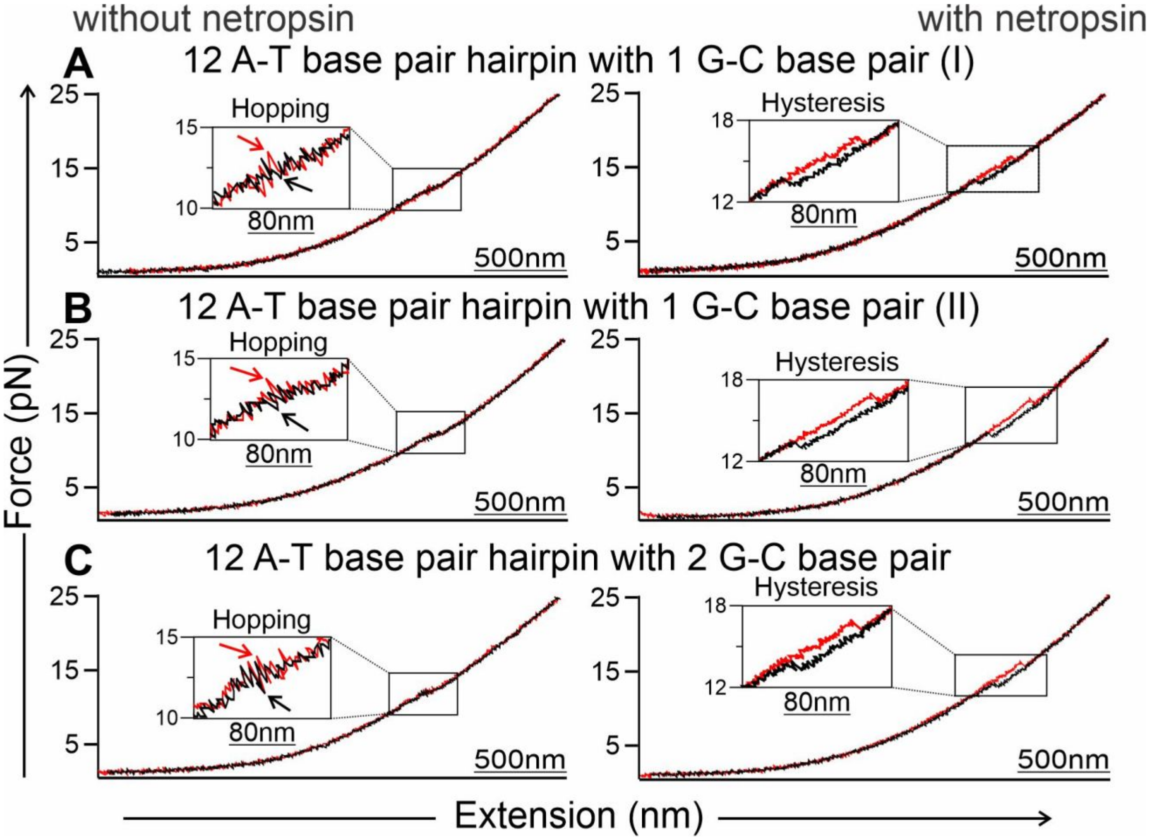

Figure S10. Typical force-extension traces for (A) 12 A-T base pair hairpin with 1 G-C base pair (I), (B) 12 A-T base pair hairpin with 1 G-C base pair (II), and (C) 12 A-T base pair hairpin with 2 G-C base pairs are shown. In control channel (100mM KCl, 10 mM Tris buffer, pH 7.4), hopping is observed (indicated with a dotted box, left panel, arrows indicate unfolding (red) and refolding (black)). In the target channel (same buffer with 100nM netropsin), hysteresis is observed (indicated in a dotted box, right panel). See Table S1 for hairpin sequence.

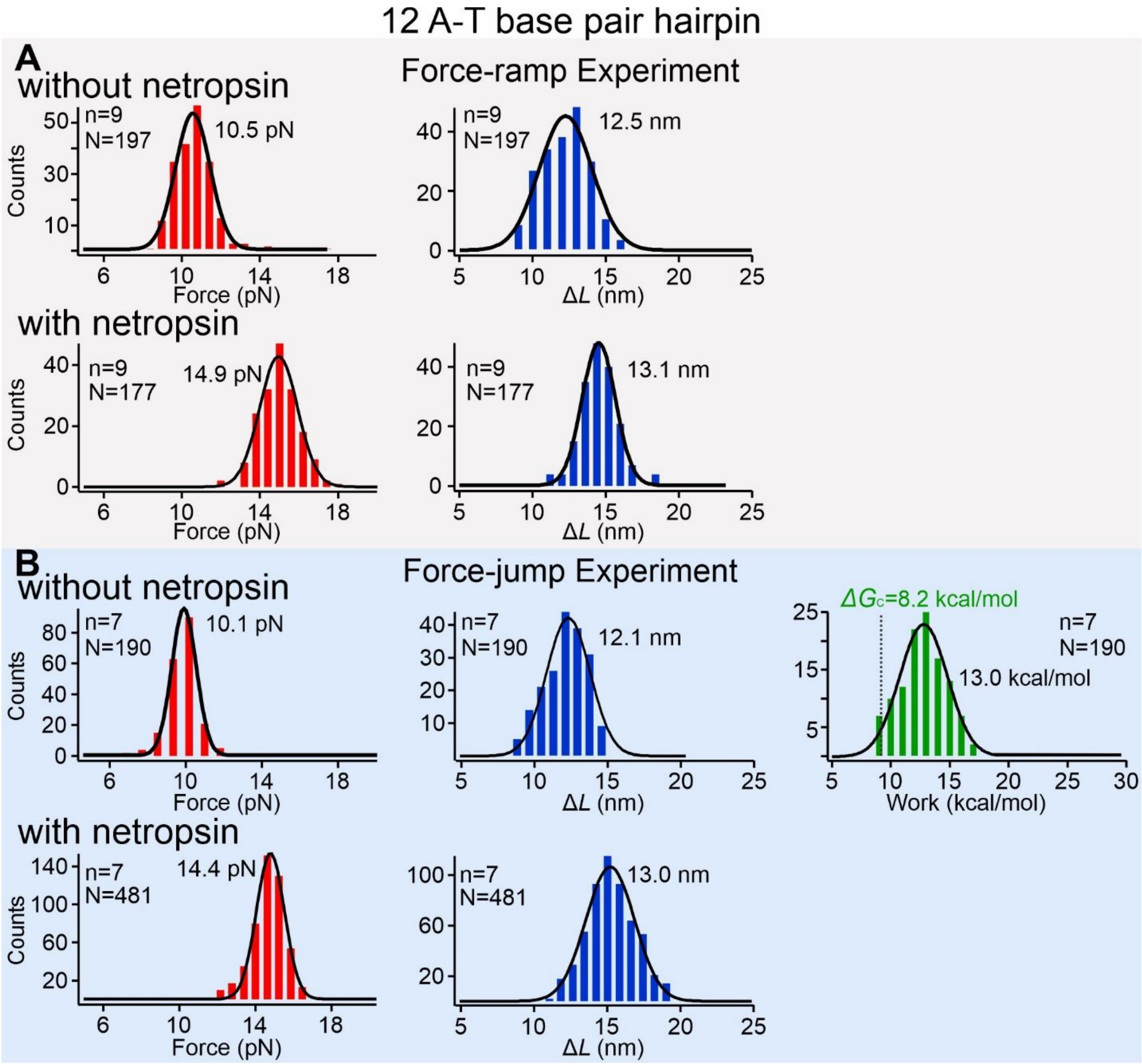

Figure S11. Histograms for unfolding force, change-in-contour-length (ΔL), and work for 12 A-T base pair hairpin obtained by (A) force-ramp and (B) force-jump experiments. For each experiment, top row shows data without netropsin and bottom row shows with netropsin. Vertical dotted lines indicate the Δ*G* values independently calculated using the Jarzynski equality, which represent the change in unfolding free energy associated with hairpin unfolding without netropsin (ΔG_C). Solid curves represent Gaussian fits and each histogram peak corresponds to the mean Δ*G* value obtained from the fitted distribution.. Numbers

indicate mean values. N and n depict the total number of data points and the number of molecules, respectively.

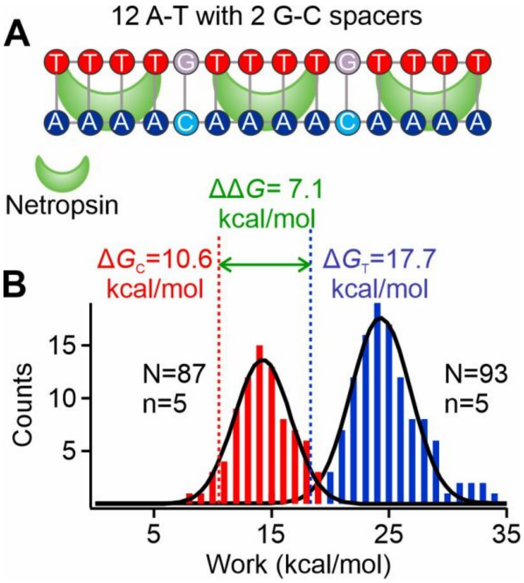

Figure S12. Free-energy analysis of netropsin binding to a 12 A-T hairpin with G-C spacers. (A) Schematic of the 12 A-T base-pair DNA hairpin containing two G-C spacers that divide the A-T tract into three independent binding sites. Green crescents indicate the netropsin molecules. (B) Unfolding work histograms obtained from force-jump experiments in the absence (red) and presence (blue) of netropsin. Vertical dotted lines indicate the change in unfolding free energies calculated using the Jarzynski equality for the control hairpin without (ΔG_C = 10.6 kcal/mol) and with bound netropsin molecules (ΔG_T = 17.7 kcal/mol). The resulting change in free-energy difference ($\Delta\Delta G$ = 7.1 kcal/mol) corresponds to the binding of three netropsin molecules. Solid curves represent Gaussian fits to the histograms. N and n depict the total number of data points and the number of molecules, respectively.

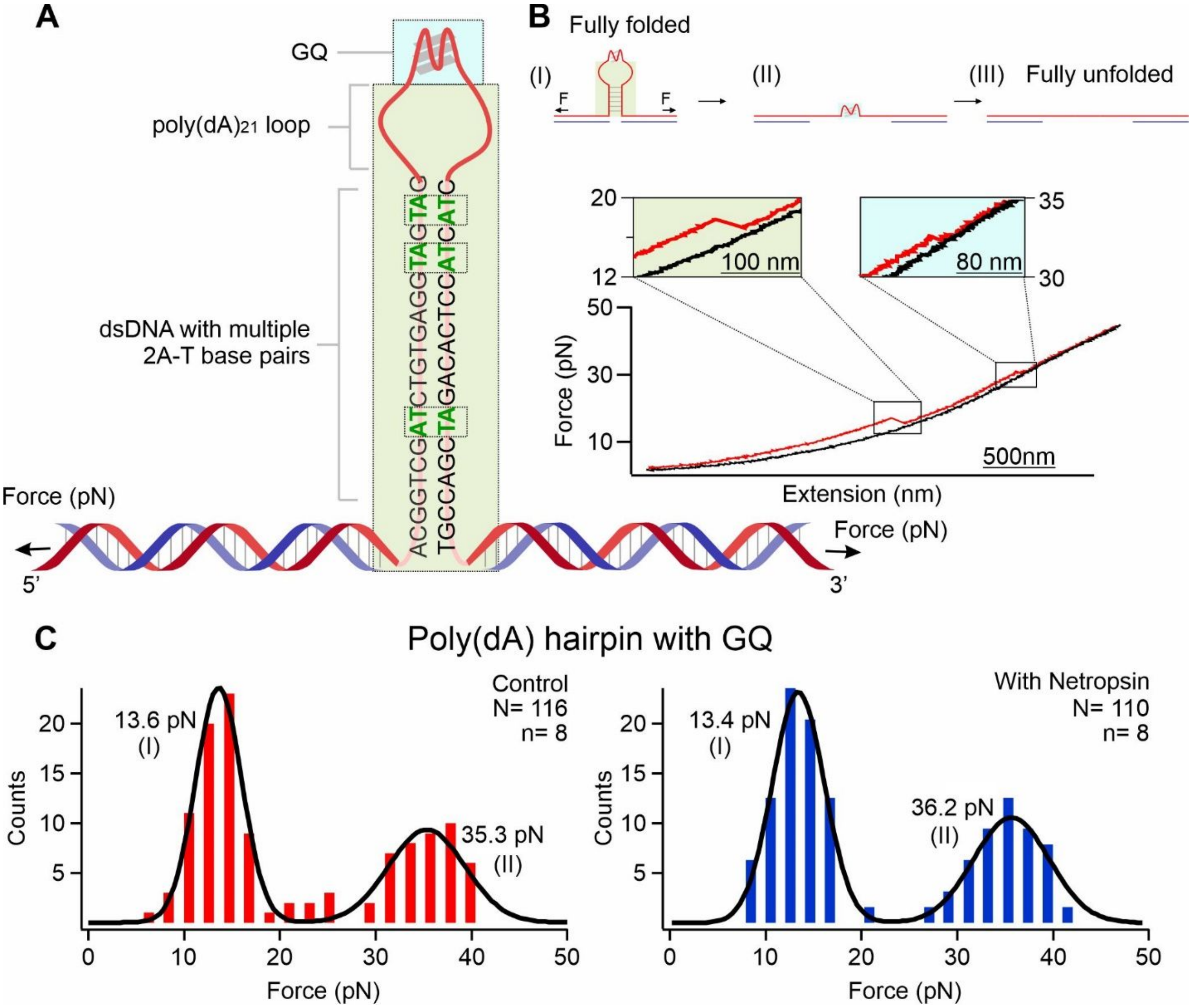

Figure S13. Figure R1. Single-molecule unfolding of a poly(dA)-G-quadruplex (GQ) hairpin in the presence and absence of netropsin. (A) Schematic of the DNA construct used in optical-tweezers experiments, comprising a GQ forming sequence (TTAGGGTTAGGGATTGGGTTAGGGTTA) connected via an internal poly(dA)$_{21}$ loop to a double-stranded DNA stem containing multiple A-T base pairs, with force applied to the 5' and 3' ends (full sequence provided in SI Table S1). (B) Representative force-extension curves showing distinct unfolding features arising from specific elements of the hairpin, where feature (I) corresponds to unfolding of the A-T-rich stem and poly(dA)$_{21}$ loop and (II) corresponds to the G-quadruplex unfolding, as indicated by the schematics above each trace. Insets highlight the corresponding force signatures. (C)

Unfolding-force histograms for the poly(dA)-GQ hairpin in the absence of netropsin, showing two distinct populations corresponding to features I and II, centered at ~13.6 and ~35.3 pN, respectively. (D) Corresponding histograms in the presence of netropsin, with similar peak positions (~13.4 and ~36.2 pN for features I and II, respectively). Solid curves represent Gaussian fits. N and n denote the total number of unfolding events and the number of molecules analyzed, respectively.

S8. Free energy difference and dissociation constant calculation.

Jarzynski's equality (7) (equation S2) was used to calculate the changes in free energy of unfolding (ΔG_{unfold}) using the works associated with unfolding events with *(ΔG_T)* and without (ΔG_C) ligand,

$\Delta G_{unfold} = -k_B \mathrm{T} \ln \sum_{i=1}^{N} \frac{1}{N} \exp\left(-\frac{w_i}{k_B T}\right)$ ....(equation S2),

where k_B is the Boltzmann constant, *T* is the absolute temperature, *N* is the number of experimental repetitions, and W_i is the non-equilibrium work done to unfold the DNA hairpins.

Work done was calculated using the equation S3,

$W_i = \sum_{i=1}^{N} (F_i \Delta x_i)$ ....(equation S3),

where *F* and Δx are the unfolding force and the change in end-to-end distance due to mechanical unfolding of the structure, respectively.

The difference in free energy change between the ligand bound and unbound states ($\Delta\Delta G$) was calculated using equation S4 for all of the hairpins containing different A-T repeats to estimate the number of bound netropsin molecules(8),

$\Delta\Delta G = \Delta G_{\mathrm{T}} - \Delta G_{\mathrm{c}}$ ....(equation S4).

The dissociation constant was then calculated using equation S5 (8),

$\Delta\Delta G = -RT ln K_d$ ....(equation S5),

where *R* is the gas constant, *T* is absolute temperature

S9. Table S2. ΔΔG for all hairpins and their respective dissociation constants (K_d).

A-T Repeats	ΔΔ*G* (kcal/mol)	K_d (M)
4	2.5	1.47×10^{-2}
8	5.2	1.59×10^{-4}
12	7.1	3.60×10^{-6}
13	6.5	1.86×10^{-5}
14	7.0	7.99×10^{-6}
15	6.5	1.83×10^{-5}
16	7.6	2.48×10^{-6}
17	10.7	1.42×10^{-8}
18	9.9	5.48×10^{-8}
20	10.9	8.88×10^{-9}

S10. References

1. Pandey, S., Xiang, Y., Walpita Kankanamalage, D.V.D., Jayawickramarajah, J., Leng, Y. and Mao, H. (2021) Measurement of Single-Molecule Forces in Cholesterol and Cyclodextrin Host–Guest Complexes. *The Journal of Physical Chemistry B*, **125**, 11112-11121.

2. Mao, H. and Luchette, P. (2008) An integrated laser-tweezers instrument for microanalysis of individual protein aggregates. *Sens. Actuators, B*, **129**, 764-771.

3. Yu, Z. and Mao, H. (2013) Non-B DNA structures show diverse conformations and complex transition kinetics comparable to RNA or proteins — a perspective from mechanical unfolding and refolding experiments. *Chem. Rec.*, **13**, 102-116.

4. Baumann, C.G., Smith, S.B., Bloomfield, V.A. and Bustamante, C. (1997) Ionic effects on the elasticity of single DNA molecules. *Proc. Natl. Acad. Sci. USA.*, **94**, 6185-6190.

5. Dhakal, S., Cui, Y., Koirala, D., Ghimire, C., Kushwaha, S., Yu, Z., Yangyuoru, P.M. and Mao, H. (2013) Structural and mechanical properties of individual human telomeric G-quadruplexes in molecularly crowded solutions. *Nucleic Acids Res.*, **41**, 3915-3923.

6. Koirala, D., Punnoose, J.A., Shrestha, P. and Mao, H. (2014) Yoctoliter thermometry for single-molecule investigations: A generic bead-on-a-tip temperature-control module. *Angew. Chem. Int. Ed. Engl.*, **53**, 3470-3474.

7. Jarzynski, C. (1997) Nonequilibrium Equality for Free Energy Differences. *Phys. Rev. Lett.*, **78**, 2690 - 2693.

8. Koirala, D., Dhakal, S., Ashbridge, B., Sannohe, Y., Rodriguez, R., Sugiyama, H., Balasubramanian, S. and Mao, H. (2011) A Single-Molecule Platform for Investigation of Interactions between G-quadruplexes and Small-Molecule Ligands. *Nat. Chem.*, **3**, 782-787.
